# How ‘Neural’ is a Neural Foundation Model?

**Published:** 2026-01-29

**Authors:** Johannes Bertram, Luciano Dyballa, T. Anderson Keller, Savik Kinger, Steven W. Zucker

**Affiliations:** 1 University of Tubingen, Germany; 2 School of Science & Technology, IE University, Madrid, Spain; 3 The Kempner Institute for Natural and Artificial Intelligence, Harvard University, Cambridge MA, USA; 4 Department of Computer Science, Yale, New Haven CT, USA; 5 Department of Biomedical Engineering, Yale, New Haven CT, USA; 6 Wu Tsai Institute, Yale, New Haven CT, USA.

## Abstract

Foundation models have shown remarkable success in fitting biological visual systems; however, their black-box nature inherently limits their utility for understanding brain function. Here, we peek inside a SOTA foundation model of neural activity (Wang et al., 2025) as a physiologist might, characterizing each ‘neuron’ based on its temporal response properties to parametric stimuli. We analyze how different stimuli are represented in neural activity space by building *decoding manifolds*, and we analyze how different neurons are represented in stimulus-response space by building *neural encoding manifolds*. We find that the different processing stages of the model (i.e., the feedforward *encoder*, *recurrent*, and *readout* modules) each exhibit qualitatively different representational structures in these manifolds. The *recurrent* module shows a jump in capabilities over the *encoder* module by “pushing apart” the representations of different temporal stimulus patterns. Our “tubularity” metric quantifies this stimulus-dependent development of neural activity as biologically plausible. The *readout* module achieves high fidelity by using numerous specialized feature maps rather than biologically plausible mechanisms. Overall, this study provides a window into the inner workings of a prominent neural foundation model, gaining insights into the biological relevance of its internals through the novel analysis of its neurons’ joint temporal response patterns. Our findings suggest design changes that could bring neural foundation models into closer alignment with biological systems: introducing recurrence in early encoder stages, and constraining features in the readout module.

## 1. Introduction

Viewed in the large, deep neural networks are intriguing mouse visual system models, learning to predict neural responses directly from visual input (Cowley et al., 2023; Ustyuzhaninov et al., 2022; Huang et al., 2023; Averbeck et al., 2006; Qazi et al., 2025; Li et al., 2023); recent foundation models even generalize beyond training data (Li et al., 2023). Viewed in the small, Representational Similarity Analysis (RSA) (Kriegeskorte et al., 2008) shows that many units reflect properties (e.g., orientation selectivity) resembling biology (Conwell et al., 2021; Qazi et al., 2025). However, despite this progress, questions arise about whether pairwise unit activity in artificial networks agrees with biological data (Liscai et al., 2025). Moreover, input/output maps remain incomplete (normalized response correlation ceilings near 70% (Wang et al., 2025)), raising questions about robustness. In effect, response correlation measures how well input drives the system to the correct output; it ignores the inverse question of how output ambiguity obscures the input. One must consider both “forward” and “inverse” mappings. Such issues are classical: control theory teaches that, without a perfect model, one must “look inside the box” to achieve identifiability (Åström, 2012). We do just this with the Foundation Neural Network (FNN) (Wang et al., 2025). Without this, we cannot guarantee robust, generalizable behavior, especially on out-of-distribution data, to confidently build brain hypotheses using the FNN. The FNN was selected as it was trained on MICrONS—the largest functional connectomics dataset of the mouse visual system (Bae et al., 2025)—using naturalistic videos across multiple animals, providing the SOTA in modeling.

**Figure 1.**
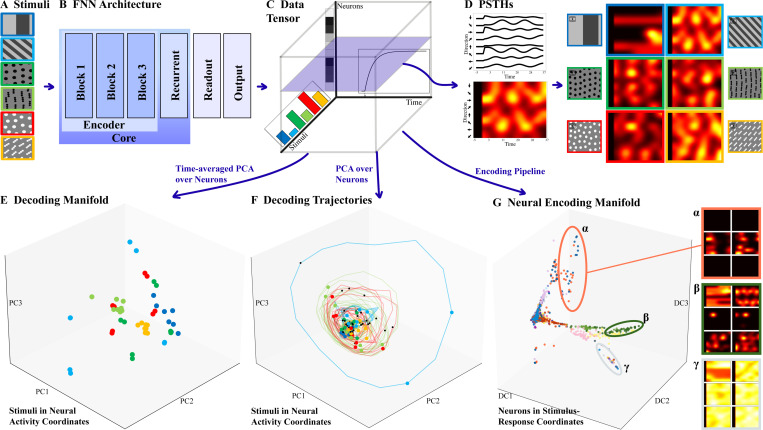
Approach and manifolds analysis **A** Stimulus ensemble provides input. **B** FNN consists of multiple encoding blocks, modeled as convolutional layers, followed by recurrent and readout/interpolation layers. **C** The tensor of data, containing the response (in time) of each sampled unit to the stimulus ensemble. **D** PeriStimulus Time Histogram: The response (instantaneous “firing rate”) of a single unit/neuron to a stimulus pattern drifting in each of 8 different directions. The curves are redrawn as an image, with brightness corresponding to activity. A plane through the data tensor shows the PSTHs for each of the 6 stimulus classes, drifting in all directions. **E** Decoding manifold, plots the total activity for each stimulus in PCA-reduced neural coordinates. Colors correspond to stimulus classes in **A**. **F** The time evolution of each stimulus presentation, plotted in PCA-reduced neural coordinates for the early encoder layer. Note the nested, periodic trajectories indicating a stimulus drifting over a receptive field filter. **G** Encoding manifold plots individual units/neurons in stimulus/response coordinates. Note the clustering of units with similar responses across the ensemble.

**Figure 2.**
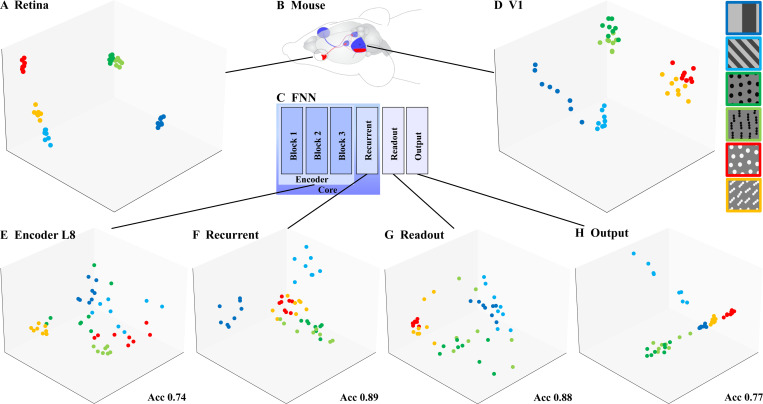
Decoding Manifolds for the mouse **(A)** retina and **(D)** visual cortex are highly clustered by stimulus (color labels shown in top-right bar) supporting decoding (i.e., reading out the stimulus from neural responses) in both cases. By contrast, the FNN is most clustered at the recurrent and readout stages **(E–H)**. Acc: classification accuracy for that layer (see Table 1). Notice how the encoder (first stage in the FNN) differs significantly from the retina (first stage in the visual system); on the other hand, the recurrent layer is most analogous to V1.

FNN consists of multiple stages (Figs. 1B, 5) and millions of units, making analyses beyond pairwise interactions—such as higher-order statistics—prohibitive. To “look inside,” we use three neuroscience techniques to: (1) evaluate how network state represents stimuli, i.e., how stimuli relate in global neural coordinates (Fig. 1E); (2) show how units relate functionally when stimulus-driven (Fig. 1G), i.e., how they encode information; and (3) observe how dynamics evolve during processing, i.e., how global neural state changes in time (Fig. 1F). The first two techniques yield manifolds characterizing *forward* and *backward* mappings, while the third yields trajectories; all are compared against biology. Briefly, while the FNN learned a forward map reasonably well, it processes stimuli differently from the mouse, making it only a “partial digital twin” dynamically. Importantly, our manifolds identify where disparities lie.

In more detail, (1) we build *neural decoding manifolds* (Chung & Abbott, 2021), in which trials are embedded in the space of neural activity coordinates (Fig. 1E), then dimensionality-reduced using Principal Component Analysis (PCA) (Cunningham & Yu, 2014). Typically, trials involving the same stimulus cluster together, facilitating a read-out of the brain’s state. (2) To switch from trials to neurons, we build *neural encoding manifolds* (Fig. 1G) (Dyballa et al., 2024a) in which each point is a neuron in the space of stimulus-response coordinates, dimensionality-reduced using tensor factorization (Williams et al., 2018). Proximity between neurons in an encoding manifold denotes similar responses to similar stimuli; i.e., groupings of neurons that are likely to share circuit properties. For a review of classic encoding/decoding in neuroscience, see (Mathis et al., 2024). Finally, (3) the relationship between these two manifolds is captured by the temporal evolution of each neuron’s activity for each stimulus trial. Recalling that a ‘neural computation’ can be viewed as the result of a dynamical system in neural state space (Hopfield, 1984), we plot these both as PeriStimulus Time Histograms (PSTHs, Fig. 1D) and as streamline traces (decoding trajectories, Fig. 1F). While streamline representations have been used previously for decision tasks (Duncker & Sahani, 2021) and the motor system (Churchland et al., 2012; Safaie et al., 2023), we note: (i) the activity integral along such *decoding trajectories* (Fig. 1F) defines the decoding manifold, while (ii) shared tubular neighborhoods (developed below) specify position in the encoding manifold. These three perspectives enable us to investigate different aspects of alignment: (1) Decoding manifolds reveal whether the model maintains stimulus separability like biology; (2) Encoding manifolds reveal whether functional topology of neurons is brain-like; (3) Trajectories reveal whether the model performs computations through brain-like dynamics. Critically, a model could succeed at one level while failing at others. We use modeling tools available online, stimuli similar to those used in FNN’s original training (Wang et al., 2025), and add naturalistic flow stimuli used in mouse physiology (Dyballa et al., 2018) (Fig. 1A).

### Prior Work.

There is an extensive literature on modeling biological neural responses (Averbeck et al., 2006; Ustyuzhaninov et al., 2022; Qazi et al., 2025), including other foundation models (Zhang et al., 2025; Azabou et al., 2023; Ryoo et al., 2025; Ye et al., 2023; 2025). We highlight that compared to these other approaches, the FNN is concerned with predicting neural activity from input videos. The FNN is an example of a data-driven predictive model (Klindt et al., 2018; Turishcheva et al., 2024; Nellen et al., 2025) with Gaussian readout (Lurz et al., 2021) that interprets the readout as per-neuron basis functions with individual readout weights. The readout thus provides an encoding embedding of biological neurons. For comparability, we use our encoding method to compare the embeddings of biological neurons and individual readout neurons. Different loss functions have been used (Nayebi et al., 2023; Bakhtiari et al., 2021; Shi et al., 2022), and others have studied decoding manifolds (Froudarakis et al., 2020; Beshkov & Tiesinga, 2022; Beshkov et al., 2024), focusing on topological properties. For a recent general review, see Doerig et al. (2023). Some studies are supportive of modeling brains with deep networks (Kriegeskorte, 2015; Yamins et al., 2014; Margalit et al., 2024), while others raise questions (Serre, 2019a). For the reasons stated above we focus on the FNN.

To our knowledge, this is the first time all three of the encoding and decoding manifold techniques have been utilized together for analysis of a perceptual system; i.e., toward *interpretability* for a foundation model. Interpretability is a rapidly evolving field for analyzing large language models (Elhage et al., 2021; Bricken et al., 2023; Skean et al., 2025), vision models (Simonyan et al., 2014; Olah et al., 2017), and recurrent models (Krakovna & Doshi-Velez, 2016). This field has been connected to neuroscience, arguing that both aim to understand complex intelligent black boxes (Kar et al., 2022; Tolooshams et al., 2025; He et al., 2024; Mineault et al., 2025). It aims to investigate the function of individual neurons, circuits, and modules in artificial networks, while in neuroscience it additionally focuses on the alignment between artificial models and biological systems (Kar et al., 2022). We tackle both challenges by trying to understand what functions the FNN modules fulfill and by testing alignment with biological representations.

Within this framework, we ask: *Do neural decoding and encoding manifolds reveal new insights into how foundation models represent temporal response patterns? Are their representations brain-like?* We hypothesize that each processing stage contributes distinct representational capabilities, all essential for fitting neural data. In particular, one might expect the *recurrent* module to enrich the temporal structure of representations, analogously to the cortex, and the encoder layers to resemble the retina with its limited recurrence. Following a brief description of our methods, we proceed to develop each of the manifolds in turn.

## 2. Methods

Our work makes novel use of publicly available open-source resources. Specifically, we employed the pretrained foundation model of neural activity (denoted FNN) provided by Wang et al. (2025), available here; and the stimuli and neural encoding manifold pipeline introduced by Dyballa et al. (2024a), accessible here. Below we briefly outline our methods (details in Appendix A).

### Model:

The FNN consists of five modules: perspective, modulation, encoder, recurrent, and readout. The encoder is a 10-layer convolutional network including 3D convolutions to capture temporal patterns for up to 12 timesteps. The core computation is performed by the feedforward-recurrent combination: a Conv-LSTM preceded by an attention layer. Finally, a separate readout module—trained individually for each mouse—performs interpolation on the recurrent output and a linear transformation to produce the FNN output.

### PSTH visualization:

Our stimulus set comprises 88 unique sequences of drifting square-wave gratings and optical flows moving in eight directions (Fig. 1A). These parametric stimuli elicit activity patterns in the FNN similar to the original natural movie training data (Appendix Figs. 9 and 10). To visualize the responses to stimuli, we group together the model’s PeriStimulus Time Histograms (PSTH) corresponding to all flow directions of a given stimulus pattern with time on the *x*-axis and direction on the *y*-axis (Fig. 1D).

### Decoding manifolds & trajectories:

We first constructed decoding manifolds by performing PCA on the stimulus-time-averaged activity data. Therefore, the decoding manifold contains 48 points, one for each unique sequence, colored by the corresponding base-stimulus (as shown in Fig. 1A); different spatial frequencies of the same stimulus are summarized with the same color. To construct *decoding trajectories*, we treated each time step as a separate data point. We compared with biological decoding results using the experimental data from Dyballa et al. (2024a).

### Alignment metrics:

We calculated Representational Similarity Analysis (RSA) (Kriegeskorte et al., 2008), Canonical Correlation Analysis (CCA) (Raghu et al., 2017), Linear Predictivity (LP) (Yamins et al., 2014), and Dynamic Similarity Analysis (DSA) (Ostrow et al., 2023) scores (details in Appendix A.12). We introduce complementary tubularity metrics to analyze neural dynamics (see Appendix A.8)

### Encoding manifolds:

To understand the response properties of *neurons* with respect to all stimuli (rather than the representation of *stimuli* in the space of all neurons), we finally constructed *encoding manifolds*. At a high level (Fig. 6), these manifolds allow one to examine the global topology of neuronal populations based on their stimulus selectivities and temporal response patterns (Dyballa et al., 2024a) (details in Appendix A.9).

## 3. Results

We built encoding and decoding manifolds, as well as decoding trajectories, for all layers of the modules considered in the FNN. Here, we focus on the results that were most informative for interpreting the computational role of each stage of the network and for comparing the FNN representations to biological results (see Appendix for ex-tended results). The decoding manifolds assess *stimulus separability*, the encoding manifolds capture *global neuronal response similarity and topology*, and the trajectories characterize *response dynamics*. Together, these analyses provide complementary perspectives for evaluating brain alignment at the population level.

### 3.1. Decoding manifolds

The biological decoding manifolds (Fig. 2A, D) showed clear clustering by stimulus with some overlap between the related 1-dot and 3-dot stimuli. It follows that neural responses at both the retina and cortical levels can be used to “read out” the stimulus. By contrast, the first encoder layer (L1) yielded a poorly clustered decoding manifold (Fig. 1E) in which stimulus classes were mixed. This implies that the latent feature representation at this point within the FNN is not sufficient to distinguish between the different stimuli (indeed, its classification accuracy is lowest; see Table 3).

The decoding manifold for layer 8 (L8) was similar to that for L1, but with greater stimulus-specific clustering. The recurrent decoding manifold was closest to the biological data, showing more distinct clusters and greater overlap between 1-dot and 3-dot stimuli. Following this, the readout and output decoding manifolds showed weaker clustering, suggesting these stages are responsible for fitting neural data rather than enriching the model’s representations. This aligns with the classification accuracy being highest for the recurrent stage and dropping again afterwards, rather differently from biology.

### 3.2. Encoding manifolds

**Figure 3.**
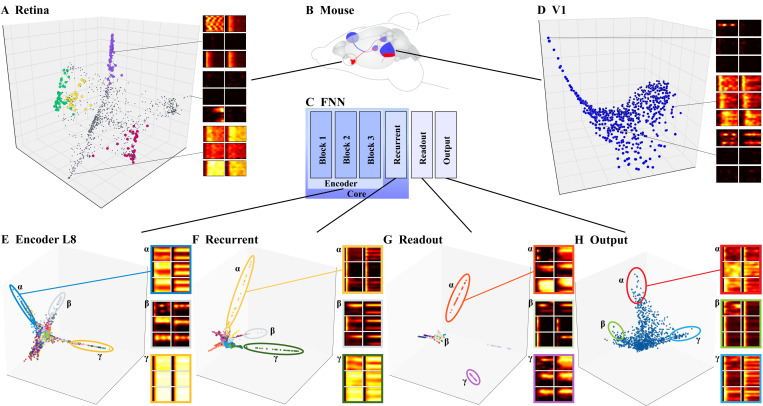
Encoding Manifolds for the mouse **(A)** retina and **(D)** visual cortex differ significantly: retina is clustered and cortex is continuous. Example PSTHs show how functionality varies smoothly in cortex but not in the retina. **(E)** The encoder stage showed a distinct arm of orientation-selective units (*α*), which are compatible with biological results, and another of intensity-based units (*γ*), which are not. **(F)** The recurrent stage showed many direction-selective units, but the following **(G)** readout stage was the most clustered among all stages. This “bottleneck” layer is then interpolated to a continuous **(H)** output layer. While the topology of this final layer is similar to that of biological visual cortex, the responses of individual units (PSTHs) are not.

The encoding manifolds were even more revealing about differences between the mouse and FNN. Replotting data from Dyballa et al. (2024a), we start with the retinal manifold (Fig. 3A). The neurons form clear clusters, each one with distinct response patterns (PSTHs) that corresponded to known retinal ganglion cell types. By contrast, the V1 encoding manifold is continuous, with smooth transitions in response patterns as it is traversed. See Dyballa et al. (2024a) for further discussion.

The encoding manifold for L1 (Fig. 1G) revealed that most neurons belonging to the same feature map (points with the same color label) formed contiguous clusters, or regions, over the manifold; this was not entirely surprising given the weight-sharing property of these convolutional layers. Nevertheless, several feature maps were found mixed into the same “arm” (labeled *β*). Examining the response patterns (PSTHs) of these neurons in detail, we observed strong, continuous activity across the entire trial duration with no selectivity for directions or stimulus classes. There was no biological counterpart to this type of neurons.

We now move on to the late-stage encoder layer, L8 (Fig. 3 E). Its encoding manifold again showed grouping by FNN feature maps, but with more mixing than in L1. We emphasize that the non-selective groups of neurons with high activity (labeled as *β* in Figs. 1E and 3E) were a significant departure from what is found in biological networks: in the retina, there are no such non-selective neurons. Although low selectivity has been observed in cortex, it is restricted to inhibitory (inter)neurons and continuously mixes with other, more selective responses; they do not segregate into an arm or cluster (Dyballa et al., 2024a).

The *recurrent* module was qualitatively different. Its encoding manifold showed that different regions exhibited distinct selectivity and temporal response patterns, as evidenced by their PSTHs (Fig. 3F). Furthermore, although segregation by feature map was still present, there was no longer a cluster of neurons with no selectivity; instead, the highlighted *β* group showed selectivity for particular directions or orientations, as is typical in biological visual neurons (e.g., PSTHs in Fig. 3D).

The final stages of the network—the *readout* and *output* layers—were again different. The encoding manifold for the readout layer analyzes the intermediate readout neurons in stimulus-response space, not the final biological output neurons. It was highly disconnected (Fig. 3G), with each cluster corresponding almost exclusively to neurons sampled from a single feature map. Each feature map exhibited a distinct response pattern that was invariant across its neurons. Compared to this, the biological results (e.g., Baden et al. (2016); Dyballa et al. (2024a)) showed more variability within functional cell “types”, even in the retina. Curiously, and despite this intra-map uniformity, the large number of feature maps (see PSTHs) and the rich dynamics within each one, somehow enable the *output* to represent the complex behavior of neurons (Fig. 3H). These behaviors are captured in the FNN output via a linear combination of *readout* features. Since classification accuracy has declined slightly at this stage (Supplemental Fig. 8), but orientation and direction selectivity agree (Supplemental Fig. 10), we conjecture that these dynamics interpolate the spiking activity individually for each mouse data used as input. The smooth manifold aligned most closely with the biological V1 manifold (Fig. 3D), although the large number of transient responses in the FNN did not match what was found in V1 (across different animals, scans, and sampling procedures).

### 3.3. Decoding trajectories

**Figure 4.**
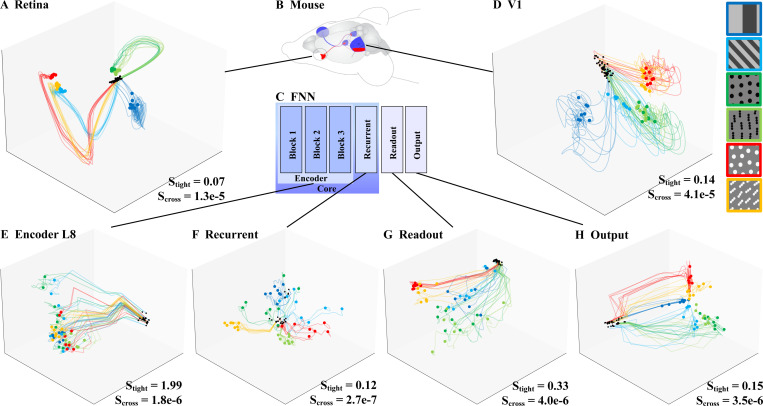
Decoding Trajectories in the retina **(A)** and V1 **(D)** show the development of neural activity dynamics into stimulus tubes. The encoder **(E)** shows only a non-selective increase in activity (see also Figure 16) rather than stimulus-dependent tubes. From the recurrent stage onward **(F–H)**, tubular trajectories similar to those seen in biological data are present. The tubularity metrics quantify this phenomenon (*S*_*tight*_), and also highlight a lack of complexity in FNN activity compared to the biological data, reflected in their lower crossings values (*S*_*cross*_).

The encoding manifolds revealed functional differences between FNN and biology: both in the topology of the neuronal organization, and in the PSTHs i.e. temporal responses for multiple stimulus classes. This motivated a direct analysis of the population response dynamics. The biological trajectories showed stimulus-dependent development of activity (Fig. 4A,D). They formed segregated, stimulus-dependent bundles whose temporal dynamics allowed linear separability during much of the trial’s time course. Here, V1 activity showed more bundles and less collinear development of trajectories. This indicates a higher complexity of response patterns in V1 compared to the retina.

Turning to FNN, the decoding trajectories for L1 revealed that periodic stimuli were represented as loops (Fig. 1F). This was likely due to the translation equivariance of the convolutional layers used in the encoder stage, which pre-served the circular geometric structure of these stimulus sequences (Cohen & Welling, 2016). However, we saw that these loops could take on many different forms (such as that for the high spatial frequency gratings, shown in light blue), influenced by the responses of particular groups of neurons to each stimulus. Layer 8, by contrast, showed stimulus-independent temporal decoding trajectories (Fig. 4E). Our analysis of removing the intensity arm from the encoding manifold showed that this temporal development of activity could be attributed to an non-selective increase in intensity during the first timesteps (Supplemental Fig. 16). Without the intensity arm, L8 has highly stationary neural activity. Thus, despite temporal convolutions, the FNN feedforward encoder appears to lack biologically plausible stimulus-dependent temporal patterns and primarily reports features present in the input, with varying intensities.

The recurrent module showed a qualitative change in decoding trajectories compared to the encoder (Fig. 4F). Similarly to the biological results, tubular temporal patterns were present at the *recurrent* stage. Still, the organization of decoding trajectories was noticeably more entangled than both retina and V1 (compare with Fig. 4A,D). This phenomenon was quantified using *tubularity* metrics based on the geometry of the observed decoding bundles (see Methods). The tightness scores were comparable between biological and FNN data from the recurrent stage onward (Fig. 4, Table 4). The retinal trajectories were the tightest, while V1 and FNN trajectories showed more expanded cones of trajectories. In particular, the FNN readout trajectories were less tight because they linearly spread out from the origin. The recurrent trajectories were also spread out, but retained a tight stimulus-dependent organization towards the end of the time frame. The tightness score for trajectories from the output stage was difficult to interpret: the predominance of transient responses caused a convergence towards a common point of low activity, which might bias the tightness score to be lower.

A more pronounced difference was observed in the crossings scores. Biological trajectories exhibited more crossings than those of the FNN, despite their tight tubular development (p *<* 0.005, Bonferroni-corrected, for all layers). These crossings occurred toward the end of the time frame, when the activity settled into a steady state. One possibility is that biological recordings contain inherently more noise, which could artificially lead to more crossings. However, the noise observed toward the end of the biological trajectories is of similar magnitude as the overall tube diameter. If measurement noise were the only cause, we would expect less coherent (tubular), more erratic (noisier) trajectory development already at earlier time steps. A second possibility is that the crossings reflect genuine neural dynamics captured in the data, suggesting that biological systems exhibit more complex temporal processing than FNN. Modulatory phenomena such as clique-like interactions (Miller & Zucker, 1999) or traveling wave activity (Pitts & McCulloch, 1947; Milner, 1974; Keller et al., 2024) could generate these apparent fluctuations. These results indicate that while parts of the FNN reproduce certain aspects of biological temporal structure (such as tubular structure), it is not yet capable of fitting the full intricacies of neural dynamics observed in biology.

The readout and output stages exhibited tubular trajectories that were less well separated than those observed in retina and V1 (Fig. 4G,H), consistent with the less clustered organization seen in the decoding manifolds. In the output trajectories, the bias towards transient responses was clearly visible as all trajectories originated from a common point (black, high activity) and converged toward a shared low-activity point via different paths.

### 3.4 Representational alignment metrics

To validate the results of our manifold analysis, we quantified the representational alignment of the FNN with both V1 and retina using standard alignment metrics from the literature (Kriegeskorte et al., 2008; Raghu et al., 2017; Yamins et al., 2014; Ostrow et al., 2023). We found that our result of the recurrent module being most aligned with biology in terms of decoding analysis was supported by these metrics (see Tables 1, 5). The DSA metric (Ostrow et al., 2023), while correctly showing higher values for tubular dynamics in the recurrent stage and after, wrongly predicted high alignment between the FNN’s L1 and the biological data. This is likely due to tubular trajectories arising for entirely different reasons (i.e., local stimulus periodicity). Moreover, smoothness and neuronal responses (PSTHs) in the encoding manifold showed a clear misalignment between the FNN’s recurrent stage and V1. This relationship was not captured by the standard metrics, underscoring the need for our analysis at the population level.

## 4. Discussion

Decoding manifolds and trajectories allow us to assess whether networks achieve comparable degrees of stimulus representation and separability. Encoding manifolds, on the other hand, evaluate at a global level how the responses and global organization of individual neurons compare to those in biological systems; in other words, whether the FNN and biological networks employ similar encoding mechanisms to produce similar outputs. Finally, decoding trajectories serve as a surrogate for *computation*, reflecting the dynamics of activity over the neural state space (cf. (Hopfield, 1984)). Our analysis of the FNN revealed an increasing richness of representation up to the *recurrent* module (cf. Hoeller et al. (2024); see also contrasts with Xu et al. (2023); Nayebi et al. (2023); Froudarakis et al. (2020)). However, most PSTHs lacked the characteristic temporal response profiles observed in biological recordings (Ringach et al., 2016; Ko et al., 2011). Since the FNN was trained to predict neural spike trains, classification behavior evolved implicitly (cf. Table 3)). Thus, it is plausible that the recurrent features are sufficiently complex for robust feature representation and that the subsequent modules serve to fit the neural data rather than to provide additional biologically meaningful computations.

However, the highly clustered topology of the latent representation observed in the *readout* module was not consistent with that of the retina or cortex (cf. Baden et al. (2016); Dyballa et al. (2024a), nor with those of higher visual areas (cf. Glickfeld & Olsen (2017); Dyballa et al. (2024b); Yu et al. (2022)). Nevertheless, the rich dynamics within each feature map (as evident in the PSTHs), together with their large number, seem to enable the output layer to capture the complex response patterns of neurons, resulting in the network’s strong performance in predicting neural activity. Still, it is somewhat surprising that such biologically realistic outputs are produced at the FNN’s output through a simple linear combination of readout features—one would expect the fitting of neural activity to occur throughout the entire network, rather than as a separate appendage module.

Our analysis pipeline was validated by its overall agreement with commonly used alignment metrics (Kriegeskorte et al., 2008; Yamins et al., 2014; Raghu et al., 2017; Ostrow et al., 2023) in predicting the closest alignment at the recurrent stage. However, the reliability of such metrics has been questioned in the recent literature (Schaeffer et al., 2025; Anonymous, 2025; Bowers et al., 2023; Lampinen et al., 2025; Dujmovic et al., 2024; Serre, 2019b). Beyond this high-level alignment, our analysis also exposed some limitations of these alignment approaches, such as with the DSA metric (Ostrow et al., 2023). This highlights the advantage of our manifold-based framework over simple metric inspection: it provides a deeper understanding of the model’s internal computations and representations. Tubularity was developed as a descriptive, data-driven characterization of population-level temporal organization. Rather than constituting an optimality principle for model design, it highlighted a salient structural property empirically present in biological recordings that was absent in early FNN layers.

### Future architecture improvements:

Our findings suggest actionable insights for bringing foundation models into closer alignment with biological systems. (1) Coupling feature extraction with temporal dynamics: In biological systems, feature extraction and the development of temporal response dynamics occur simultaneously. Enforcing temporal dynamics in the early layers could enable more adequate modeling of the rich retinal dynamics. The FNN uses two temporally aware mechanisms in the recurrent module: attention and recurrence. We argue that recurrence, rather than attention, is the critical mechanism, as the FNN without attention yielded equal or better performance (Wang et al., 2025). Although our analysis was limited to the published attention-based version, we propose introducing early-stage recurrence that mimics amacrine cell connectivity in the retina (Marc et al., 2014). (2) Revising the readout stage: The current Gaussian readout layer (Lurz et al., 2021) combines a large number of feature maps through a single linear combination step, producing unrealistically distinct feature representations. Enforcing mixed features while reducing their number to better reflect biological cell type diversity (Bae et al., 2025) could push the representation towards smoother and more biologically realistic manifolds.

### Limitations:

Our analysis used a single foundation model, due to the limited availability of other video-based foundation models of neural activity over time. Moreover, we worked with a restricted set of stimuli (see Methods) to ensure comparability with biological data. However, there is evidence that these stimuli exercise much of the mouse visual cortex Dyballa et al. (2018), so they provide at least a necessary component for out-of-sample examination. Moreover, we show that these stimuli elicit activity patterns in the FNN similar to those evoked by the natural movies on which they were trained (Appendix Fig. 9), supporting their empirical validity. Finally, the tubularity metrics introduced here represent a novel approach for quantifying the geometry of neural trajectories. As no established methodological standards currently exist, further investigation of these metrics would be valuable. Specifically, systematic investigations of on both biological data and synthetic datasets would help assess robustness and for obtaining clear baselines. Additionally, incorporating curvature information could extend the metrics to capture additional characteristics of neural trajectories.

## 5. Conclusion

We found a rich diversity of encoding and decoding topologies in the FNN, highlighting its capability to fit complex neural data. Distinct representation patterns emerged across modules, reflecting its architecture. First, the *recurrent* module appears to learn generalizable representations of temporal stimuli, promoting uniformity and alignment, as in general self-supervised foundation models (Wang & Isola, 2022). Second, the *readout* module accounts for rich biological variability, but does so through a large number of self-similar feature maps, differing from the heterogeneous organization known in V1. Finally, the output layer achieves a continuous representation by linearly combining the readout features, ultimately enabling the network to associate spike trains with input movies *a posteriori*.

Using our novel tubularity metrics, we found that biological data exhibited strong stimulus-dependent structure in both retina and V1, whereas the FNN encoder trajectories lacked such tubularity. Only from the recurrent module onward did the FNN begin to form bundles of activity, reaching higher–though still sub-biological–levels of representational cohesion. This emphasizes the role of recurrence in generating biologically plausible temporal representations, suggesting that models may benefit from placing recurrence after a more light-weight, local encoder (e.g., emulating the amacrine connectivity in the retina (Marc et al., 2014)) and that constrain feature dimensionality to reflect biological cell-type diversity (Bae et al., 2025). While biological fidelity is not a prerequisite for achieving high predictive accuracy, digital-twin use cases require enough internal alignment to support mechanistic and interventional inference. Such designs could help bridge the gap between computational performance and biological plausibility, moving toward truly brain-aligned foundation models.

## Supplementary Material

Supplement 1

## Figures and Tables

**Table 1.** Mean representational alignment metrics. Mean of Representational Similarity Analysis (RSA), Canonical Correlation Analysis (CCA), Linear Predictivity (LP) and Dynamic Similarity An lysis (DSA). Details in Appendix A.12); individual values in Table 5.

Region	Enc L1	Enc L2	Enc L4	Enc L5	Enc L7	Enc L8	Rec	Readout	Output
Retina	0.26	0.26	0.30	0.33	0.28	0.28	**0.40**	0.34	0.34
V1	0.29	0.21	0.32	0.30	0.30	0.32	**0.53**	0.38	0.48
