# Supplementary material for: How ‘Neural’ is a Neural Foundation Model?
Source: ArXiv. 2026 Jan 29:arXiv:2601.21508v1. Preprint. [Version 1] (PMC12869402)
Supplement: Supplement 1 [file NIHPP2601.21508v1-supplement-1.pdf]

Appendix

A. Methods

A.1. Online material

Our work made use of publicly available open-source resources. Specifically, we employed the pretrained FNN model provided by Wang et al. (2025), available at https://github.com/cajal/fnn/tree/main. For the analysis of this model, we used the stimulus generation tools and neural encoding manifold construction pipeline introduced by Dyballa et al. (2024a), accessible at https://github.com/dyballa/NeuralEncodingManifolds.

A.2. FNN

The FNN consists of five modules: perspective, modulation, *encoder*, *recurrent*, and *readout* (Fig. 5). The perspective and modulation modules model the mouse's state and transform the inputs to approximate the actual visual information received. Thus, only the *encoder*, *recurrent*, and *readout* modules perform the core computation and are the focus of this work.

The *encoder* module is a 10-layer DenseNet-style convolutional encoder. Notably, it includes 3D convolutions, which in principle enable the encoder to capture temporal patterns, for up to 12 time steps into the past for later encoder layers. The *recurrent* module is optionally preceded by an attention layer and consists of a convolutional LSTM, followed by a single convolutional layer that produces its output. This feedforward–recurrent combination constitutes the core of the FNN, which is trained on all data. Finally, the *readout* module is mouse-specific: it performs an interpolation on the recurrent output followed by a linear transformation to produce the FNN output. We used the FNN readout from session 8, scan 5 as this was the exemplary scan used in the authors' tutorial. We validated findings on several other sessions and scans.

A.3. Input videos

We used the visual stimuli from Dyballa et al. (2024a), consisting of drifting square-wave gratings and optical flows moving in eight directions. The flow stimuli include oriented (lines) and non-oriented (dots) stimuli with spatial frequencies between 0.04 and 0.5 $\frac{\text{cycles}}{\text{deg}}$. This yields 88 unique input sequences with stochastic initial positions and velocities. The stimuli were scaled and cropped to fit the required FNN input shape of 144×256 pixels. This resulted in an image sequence: $\{\mathbf{x}_0, \ldots, \mathbf{x}_T\}$, where each $\mathbf{x}_i \in \mathbb{R}^{H \times W}$. Stimuli were generated using the tools available at https://github.com/dyballa/NeuralEncodingManifolds.

The FNN (Wang et al., 2025) processes 2.33-second sequences of 70 frames each, corresponding to 30 frames per second. Since in Dyballa et al. (2024a) the trials were 1.25 s long, we adapted the stimuli to contain 37 frames to maintain consistency with the FNN framework. This adaptation was performed using the hyperparameters of the stimulus generation pipeline, allowing comparable stimuli dynamically created for different lengths and number of frames.

We acknowledge a difference in the experimental setups regarding the visual field: Wang et al. (2025) used a screen distance of 15 cm, whereas the stimuli from Dyballa et al. (2024a) were originally designed for a 25 cm viewing distance. This discrepancy potentially affects the visual field transformations performed by the model's perspective module, as the visual angle subtended by the stimuli differs between the two configurations. We applied a global scaling factor of 0.7 to all stimuli to address this. This adjustment was empirically found to optimize stimulus discriminability across network layers, effectively bridging the geometric gap between the training and analysis domains.

A.4. Data sampling

Neural responses were computed using PyTorch and extracted by sampling activations from 2000 units across selected FNN layers. Within each layer, 40 feature maps were sampled. Then, 50 neurons were sampled from each feature map. Feature map sampling probabilities were calculated from the mean maximum response across all neurons within each map, while neuron sampling probabilities within each selected feature map were based on individual neuron maximum responses, biasing the sampling to include active neurons. This sampling procedure was chosen to ensure comparability to the biological results from Dyballa et al. (2024a). This sampling procedure was tested and validated in Dyballa et al. (2024a); we performed further tests with random sampling to validate this bias does not filter out relevant structures. One exemplary sampling result, showing qualitative stability of results across sampling strategies and sizes can be found in Fig 18. Increasing the sampling rate beyond 2000 units did not significantly alter manifold topology but hindered cluster separation in diffusion map analysis. The resulting tensor data had dimensions $(N \times S \times O \times T)$ with $N = 2000$ neurons, $S = 11$ stimulus types, $O = 8$ orientations and $T = 37$ time steps. For manifold construction, the optimal spatial frequency was selected (resulting in $S = 6$ stimuli) whereas for classification performance all spatial frequencies were kept. We report results from a single random seed per layer, as preliminary analysis showed consistent manifold structure across different random activity samples. These neural activation tensors served as

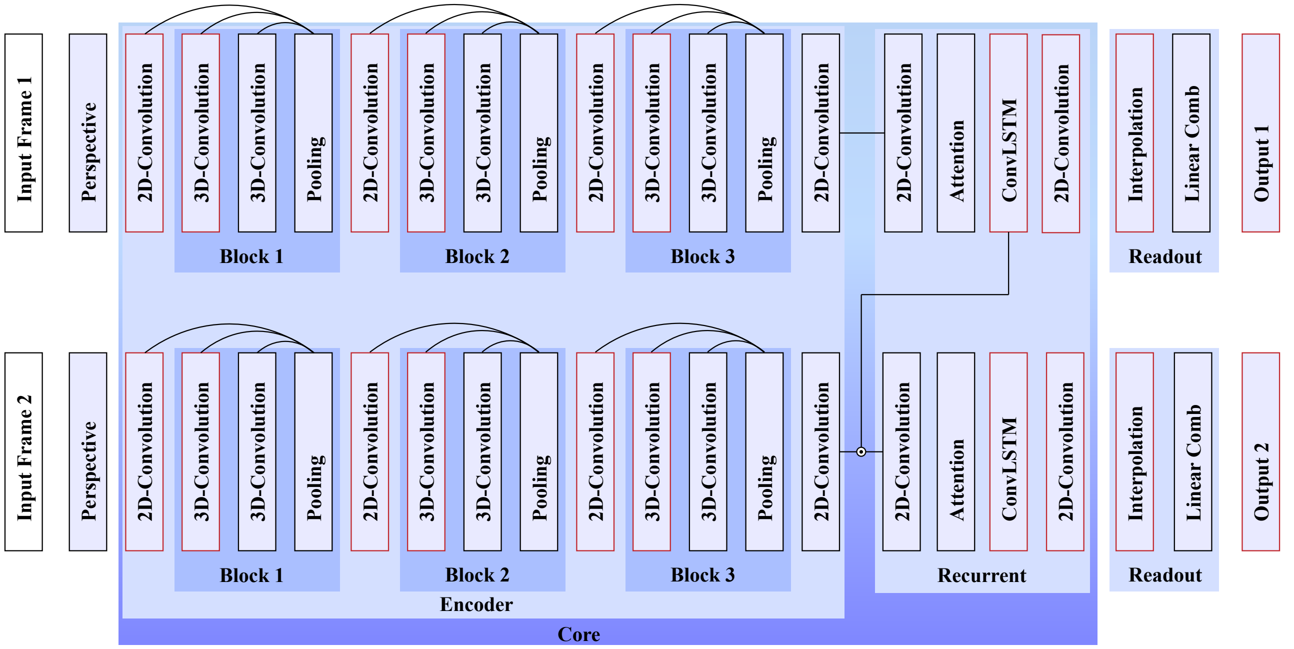

Figure 5. **FNN architecture.** Layers used for sampling are highlighted. Modulation module omitted as it has no effect for our analysis. The FNN used GeLU activations in the convulutional layers, and Tanh activations in the Recurrent module.

input for subsequent classification and manifold analysis. This sampling procedure was developed by Dyballa et al. (2024a) and tested against other sampling methods there. We also experimented with the sampling procedure, finding that random sampling and increased sampling rate did not introduce qualitative changes to the manifolds.

A.5. Stimulus adequacy

For every FNN layer investigated in this paper, we extracted the activation to the stimulus ensemble consisting of gratings and flows (see Section A.3) as well as to a 100-second-long natural input video from the MICrONS functional dataset (Bae et al., 2025), downloaded from s3://bossdb-open-data/iarpa_microns/minnie/functional_data/stimulus_movies/. Both stimulus sets produced similar activation magnitudes across the entire network (see Fig. 9), which shows the adequacy of the stimulus ensemble used for testing the FNN.

For orientation and direction selectivity, we followed Wang et al. (2025)’s procedure: We input directional pink noise (16 directions, 37 frames) to the model and record the output activations. Additionally, we recorded the outputs for our flow stimuli. For both datasets, we computed the Orientation Selectivity Index (OSI) and Direction Selectivity Index (DSI) and compared their distributions.

$$OSI = \frac{|\sum_\theta \overline{r}_\theta e^{i2\theta}|}{\sum_\theta \overline{r}_\theta} \tag{1}$$

$$DSI = \frac{|\sum_\theta \overline{r}_\theta e^{i\theta}|}{\sum_\theta \overline{r}_\theta} \tag{2}$$

Here, $\overline{r}_\theta$ is the mean response for angle θ. We found comparable OSI and DSI distributions (see Fig. 10).

A.6. Classification accuracy

Classification performance of the stimulus set, measured using activations, serves as a proxy for representational richness. Logistic regression is employed to assess linear separability, while k-Nearest Neighbor (k-NN) classification is used to evaluate local geometric structure for comparison with logistic regression.

Stimulus classification accuracy based on individual-layer activities was determined by training multinomial logistic regression classifiers (solver: L-BFGS) with 5-fold cross-validation (CV). Only sampled neurons were used to classify the 11 stimuli. For each layer and each time point t, two feature sets were constructed: (i) the mean activity over frames 0 to t (increasing window) and (ii) the mean activity over frames t to end (decreasing window). For comparison, K-nearest neighbor classifiers (K=3) were also evaluated using leave-one-out CV. The value K=3 was selected as the optimal neighborhood size. Leave-one-out CV was used for k-NN due to its suitability for small datasets, while 5-fold

CV was chosen for logistic regression to reduce computational requirements. Results are summarized in Table 3 and Fig. 8.

A.7. Construction of decoding manifolds

For building the *decoding manifolds*, we applied PCA (scikit-learn) to the averaged activity data. In total, the *decoding manifolds* contain 48 points, consisting of 6 stimuli and 8 movement directions each. The 6 stimuli were obtained from a majority vote of all neurons on the optimal spatial frequency eliciting higher responses. The decoding manifolds use different colors for each stimulus, as introduced in Fig. 1. Different spatial frequencies of the same stimulus are summarized with the same color. To construct *decoding trajectories*, we treated each time step as a separate data point rather than averaging across time before applying PCA. In both cases, we reduced the dimensionality to three components for visualization after verifying that further dimensions did not encode qualitatively new information. We constructed biological *decoding trajectories* using experimental data from Dyballa et al. (2024a), available at `https://github.com/dyballa/NeuralEncodingManifolds`. For the biological decoding trajectories, we did not use the additional zero-activity time step since a baseline activity level was already provided by the inter-stimulus intervals in the experiments.

A.8. Tubularity

To investigate neural dynamics, we modeled trajectories by bundling them into tubular neighborhoods around a central skeleton (Budanur, 2023). We operationalized this idea for discrete data using the tubular neighborhood theorem (Da Silva, 2008), which guarantees that smooth submanifolds admit non-intersecting neighborhoods diffeomorphic to their normal bundles. Tubularity is not a trajectory-matching metric but a population-geometry metric: it assesses the structure of collections of trajectories rather than the similarity of individual pairs.

Before calculating tubularity metrics, we standard-scale the data and apply PCA to obtain a 10-dimensional embedding, thereby speeding up the computation. While visualizations use only the first 2–3 dimensions, all metrics are calculated in the 10-dimensional space. To ensure comparability, we resampled all trajectories to length 100. For statistical analysis, we generated 100 bootstrapped samples, and using ground-truth clusters, performed Bonferroni-corrected Mann-Whitney U tests on our hypotheses.

We formalize how “tight” a group of curves is around the centerline: We reparameterize each curve by normalized arc length $u \in [0,1]$ and resample to $\{u_k\}_{k=1}^{M}$. Let $x_i(u_k) \in \mathbb{R}^D$ denote the samples and $\tau_i(u_k)$ their unit tangents. We define the *mean curve* as the pointwise average:

$$c(u_k) = \frac{1}{m}\sum_{i=1}^{m} x_i(u_k), \qquad r_i(u_k) = \|x_i(u_k) - c(u_k)\|.$$

The tightness score is calculated by averaging quantile tube radii across bins $\{I_b\}_{b=1}^{B}$ that partition $[0,1]$, using a high quantile $q \in [0.8, 0.95]$ to ensure robustness to noise. We normalize each tube’s tightness score by tube length.

$$S_{\text{tight}} = \frac{1}{B}\sum_{b=1}^{B} \text{quantile}_q\{\, r_i(u) : u \in I_b \text{ over all curves} \,\}.$$

The second quantity assessed is the uniformity of the tubes relative to one another. That is, the degree to which crossings occur in our defined bundle of curves. Tubes are considered disorganized when distinct curves pass near each other with *transverse* directions. Let $d_{ij}(u,v) = \|x_i(u) - x_j(v)\|$ and $\phi_{ij}(u,v) = 1 - \langle \tau_i(u), \tau_j(v)\rangle^2 \in [0,1]$ (large for near-orthogonal tangents). Using a Gaussian kernel $K_\varepsilon(\rho) = \exp(-\rho^2/(2\varepsilon^2))$, we softly count encounters:

$$\mathcal{X}_\varepsilon = \frac{2}{m(m-1)}\sum_{i<j}\int_0^1\int_0^1 K_\varepsilon\big(d_{ij}(u,v)\big)\,\phi_{ij}(u,v)\,du\,dv.$$

S_{tight} and S_{cross} only depend on distance, unit-tangent inner product, and arc-length. Therefore, they are invariant to translations, rotations, and re-timing. We emphasize that, for both scores, smaller values indicate more tubular curve bundles, while larger values indicate fewer tubular curve bundles.

A.9. Construction of neural encoding manifolds

At a high level, the motivation for constructing *neural encoding manifolds* is to find a space in which one can examine the global topology of neuronal populations based on their stimulus selectivities and temporal response patterns (Dyballa et al., 2024a). The neural encoding manifold is constructed in a three-step procedure. First, a 3-tensor is built with the temporal responses from each neuron for each stimulus, and decomposed using Nonnegative Tensor Factorization (details below); each component is comprised of neural, stimulus, and temporal response factors. The neural factors then serve as position coordinates, embedding the neurons into a stimulus-response framework called the neural encoding space. Second, we construct a data graph in this neural encoding space using the IAN algorithm (Dyballa & Zucker, 2023). Third, applying diffusion maps

(Coifman et al., 2005; Coifman & Lafon, 2006) to the data graph yields the manifold.

The methodological choices in our manifold construction procedure are made in accordance with Dyballa et al. (2024a), where extensive parameter analysis for biological neural data was conducted. Since *neural encoding manifolds* computed with these specific parameters represent the only available comparison for biological data from the visual system, we maintained their parameter settings to ensure direct comparability between artificial and biological neural representations. We further conducted analysis for FNN-specific parameters, such as the sampling procedure, by adapting their code to fit the FNN requirements.

A.9.1. Preprocessing

The input tensor of neuronal activity (see above) was preprocessed in several steps (using NumPy and SciPy). First, the individual responses were smoothed along the time dimension using a one-dimensional Gaussian kernel with $\sigma = 3$. Next, we grouped the stimuli into *medium* versus *high* spatial frequencies and selected the one exhibiting higher response magnitudes. The temporal responses for the 8 directions of motion were then concatenated together into a single vector. Finally, we normalized each response and rescaled it by the relative activations of the neuron. The resulting tensor $\mathbf{T}$ had shape $((N = 2000) \times (S = 6) \times (O * T = 296))$.

A.9.2. Nonnegative Tensor Factorization

Next, Nonnegative Tensor Factorization (see (Williams et al., 2018) for an overview and applications to neuroscience) was applied to our tensor $\mathbf{T}$. It was decomposed into typically 10–15 rank-1 tensors which are obtained from the outer product of three vectors each. We selected the number of components separately for each data sample based on changes in explained variance and noise, following the procedure in Dyballa et al. (2024a). The factors in each component are scaled to unit length, and their magnitudes absorbed by a scalar λ_r:

$$\tilde{\mathbf{T}} = \sum_{r=1}^{R} \lambda_r \mathbf{v}_r^{(1)} \circ \mathbf{v}_r^{(2)} \circ \mathbf{v}_r^{(3)} = [\lambda; \mathbf{X}^{(1)}; \mathbf{X}^{(2)}; \mathbf{X}^{(3)}] \quad (3)$$

For the second equality, the factor matrices $\mathbf{X}^{(k)}$ are constructed using the factor vectors $\mathbf{v}_r^{(k)}$ as columns, and the vector λ contains all individual λ_rs.

Decomposing the tensor $\mathbf{T}$ into these components is an optimization problem with the following objective function and non-negativity constraints:

$$\min_{\mathbf{X}^{(1)},\mathbf{X}^{(2)},\mathbf{X}^{(3)}} \frac{1}{2}||\mathbf{T} - \tilde{\mathbf{T}}||^2 \quad (4)$$

$$\text{such that} \quad \mathbf{X}^{(k)} \geq 0, \forall k \quad (5)$$

The resulting decomposition is interpretable: the third group of vectors, $\mathbf{v}_r^{(3)}$, describes different temporal response patterns; $\mathbf{v}_r^{(2)}$ contain information about which stimuli exhibit these response patterns; and $\mathbf{v}_r^{(1)}$ are the neuronal factors determining which neurons exhibit the response patterns characterized by $\mathbf{v}_r^{(2)}$ and $\mathbf{v}_r^{(3)}$. During decomposition, circular permutations were applied to detect patterns irrespective of the preferred orientations of specific neurons (again, this is necessary to ensure compatibility with the biological results from (Dyballa et al., 2024a)).

Using the OPT method from Tensor Toolbox (Bader et al., 2023)), we ran the decomposition 50 times (different initializations) for each number of components and dataset to ensure robust decomposition results and the choice of the number of factors, R. The manifolds were robust to small changes in R, therefore the heuristic for choosing R based on the explained variance of the decomposition outlined in Dyballa et al. (2024a) proved sufficient. For building the manifolds, we used the result with smallest reconstruction error among the 50 initializations.

A.9.3. Neural encoding space

Following Dyballa et al. (2024a), we now reformulate the above decomposition to construct the neural encoding space. By defining the diagonal matrix $\mathbf{\Lambda}$ with $\mathbf{\Lambda}_{rr} = \lambda_r$, we obtain:

$$\tilde{\mathbf{T}} = \mathbf{X}^{(1)} \mathbf{\Lambda} (\mathbf{X}^{(2)} \circ \mathbf{X}^{(3)}) \quad (6)$$

Since the first matrix, $\mathbf{X}^{(1)}$, represents the neuronal factors, we denote it by $\mathcal{N}$. Now, define a matrix $\mathbf{B}$ with columns $\mathbf{b}_{:,r}$:

$$\mathbf{b}_{:,r} = vec(\mathbf{v}_r^{(2)} \circ \mathbf{v}_r^{(3)}) \quad (7)$$

Finally, we obtain a matrix representation of $\mathbf{T}$ with respect to neuronal factors as $\mathbf{X}_{\mathcal{N}}$:

$$\mathbf{X}_{\mathcal{N}} = \mathbf{B} \mathbf{\Lambda} \mathcal{N}^T \quad (8)$$

This reformulation constructs the neural encoding space. The unit-norm basis vectors of this space are given by the

columns of $\mathbf{B}$. We define the neural matrix containing the positions of all neurons in this space as $\mathcal{N}_\lambda = \mathcal{N}\mathbf{\Lambda}$. The distances between any two neurons in this space reflect their similarity in stimulus-selective temporal response patterns. Intuitively, neurons with similar selectivity profiles and temporal dynamics should be positioned close together, while neurons with dissimilar response characteristics should be farther apart.

A.9.4. Iterated adaptive neighborhoods (IAN)

Within this neural encoding space, we construct a weighted graph of the data by inferring a similarity kernel. This is achieved using the Iterated Adaptive Neighborhoods (IAN) algorithm (Dyballa & Zucker, 2023), which infers an adaptive local kernel without the need for pre-specifying a fixed neighborhood size.

IAN first constructs the unweighted Gabriel graph for the data points. In addition, a weighted graph is constructed using a multiscale Gaussian kernel based on the discrete neighborhood graphs. Subsequently, the graph is iteratively pruned by ensuring consistency between the discrete and continuous neighborhoods. The resulting weighted graph is represented by the adjacency (kernel) matrix $\mathbf{K}$. This matrix contains similarities computed using locally tuned Gaussian kernels.

A.9.5. Diffusion maps

Diffusion Maps (Coifman et al., 2005; Coifman & Lafon, 2006) are a dimensionality reduction technique that retain distances and preserve the intrinsic geometry of the manifold. The diffusion process is based on graph Laplacian normalization from spectral graph theory.

In detail, we use the weighted graph obtained from IAN as the weighted adjacency matrix $\mathbf{K}$. The first step is to normalize and symmetrize it to produce $\mathbf{M}_s$:

$$\mathbf{d}_i = \sqrt{\sum_j \mathbf{K}_{ij} + \epsilon} \tag{9}$$

$$\mathbf{M}_s = \frac{\mathbf{K}}{\mathbf{d}\mathbf{d}^T} \tag{10}$$

This normalization ensures that nodes of high degree do not dominate the analysis. We then calculate the spectral decomposition of $\mathbf{M}_s$ with eigenvalues $\lambda_0 = 1 \geq \lambda_1 \geq \lambda_2 ...$ and eigenvectors $\boldsymbol{\psi}_i$ for $t = 1$ diffusion steps using $L = 20$ eigenvalues:

$$\mathbf{M}^t_{s,ij} = \sum_{l=0}^{L} \lambda_l^{2t} \boldsymbol{\psi}_l(i) \boldsymbol{\psi}_l(j) \tag{11}$$

Finally, from the spectral decomposition, we obtain the diffusion map with diffusion coordinates:

$$\Psi_t(i) = \begin{pmatrix} \lambda_0^t \boldsymbol{\psi}_0(i) \\ \lambda_1^t \boldsymbol{\psi}_1(i) \\ \vdots \\ \lambda_{L-1}^t \boldsymbol{\psi}_{L-1}(i) \end{pmatrix} \tag{12}$$

Plotting the data using these diffusion coordinates yields the *neural encoding manifold.*

A.9.6. Encoding manifold visualization

For visualization purposes, we optionally applied metric multidimensional scaling (MDS) to the diffusion map coordinates. This was done by computing pairwise squared Euclidean distances using the first diffusion coordinates, constructing the corresponding Gram matrix $\mathbf{G} = -0.5 * \mathbf{D}^2$, and applying kernel PCA to obtain a lower-dimensional embedding. This preserves the distance relationships from the diffusion map while combining multiple diffusion coordinates, enabling a clearer visualization of the manifold structure.

Based on the manifold topology, we selected groups of neurons to investigate via their PeriStimulus Time Histograms (PSTH). We averaged their activity across trials and constructed the PSTHs as a 2-D heatmap, where each row contains the temporal activity in response to a particular direction of motion (as displayed in Fig. 1). Additionally, we calculated the average response intensity over time for these groups and reported the s.e.m. using the shaded regions (see insets in Fig. 6A,D).

A.10. Visualizations

Interactive three-dimensional plots of the manifolds were computed using Plotly. Other plots were created with Matplotlib and TUEplots.

A.11. Minimodels

For our additional analysis in Fig. 17, we used the convolutional model introduced in Du et al. (2025). We downloaded model checkpoints from `https://github.com/MouseLand/minimodel/tree/main`. We left the manifold pipeline unchanged for this experiment and sampled activations from layer 2.

A.12. Alignment Metrics

For comparability, the biological data was downsampled to 37 time steps for all alignment metric calculations. Except for DSA, all metrics were calculated on the individual time steps and the averaged.

A.12.1. Representational Similarity Analysis (RSA)

RSA (Kriegeskorte et al., 2008) is computed by obtaining the Representational Dissimilarity Matrices (RDMs) for every time step individually via $RDM = 1 - PearsonCorrelation$. Then, based on the upper triangular values (excluding diagonals), the RSA scores are obtained from the Spearman's r (using scipy stats) between biological and artificial data.

A.12.2. Canonical Correlation Analysis (CCA)

For CCA (Raghu et al., 2017), the data was first dimensionality reduced using PCA (3 components). Then, using sklearn's CCA function, the first 3 canonical vectors were obtained and their correlations between brain and model were averaged, yielding CCA.

A.12.3. Linear Predictivity (LP)

Linearly predicting individual biological neurons from FNN data using Ridge Regression did not yield adequate scores due to the high amount of noise. Therefore, we again used PCA to obtain 3 components for brain data and 20 components for artificial data. We then fit Ridge Regression ($\alpha = 1$ to predict individual components of biological data using 40 random stimuli, and measured the average performance on the 8 heldout stimuli via R^2 (Yamins et al., 2014).

A.12.4. Dynamical Similarity Analysis (DSA)

For DSA (Ostrow et al., 2023), we again simplified data using PCA (10 components) and computed DSA scores using the DSA function from the authors. We reported inverted DSA scores ($1 - DSA$) to compare with other metrics, and Z-scores compared to a null distribution of 50 samples where the time steps of FNN data were randomly shuffled before comparing to biology.

A.12.5. Critique

The validity of these methods for comparing brains and machines has been questioned (Serre, 2019b). Schaeffer et al. (2025) argue that lower LP scores may correspond to less brain-like models, as, instead of selecting biological models, LP overfits biases in linear regression. They claim that the same holds for overfitting to other representational similarity metrics. It is unclear what brain alignment means and what the different alignment metrics truly measure (Anonymous, 2025). Metric variability can fall within individual subject variability, making clear conclusions difficult (Anonymous, 2025). Bowers et al. (2023) question the assumption of biological visual systems being optimized to classify objects. Also, they claim that differences in the features used in DNNs compared to those in the brain can lead to high similarity. Moreover, simple features can be overrepresented compared to complex features, biasing the similarity scores (Lampinen et al., 2025). Finally, Dujmovic et al. (2024) find that metrics like RSA are not robust with respect to input perturbations.

A.13. Intensity artifacts

We now move on to the late-stage encoder layer, L8 (Fig. 3 E). Its encoding manifold again showed grouping by FNN feature maps, but with more mixing than in L1. This was especially true in the poorly selective “intensity arm” of neurons, (β) which exhibited strong response (PSTHs) for all stimuli across multiple feature maps. Further investigation revealed that the intensity arm resulted from an FNN technical requirement: padding artifacts at the edges of feature maps. Such artifacts are a well-known issue in convolutional models (Alsallakh et al., 2020), and we also observed them in Du et al. (2025)'s model (Fig. 17). Sampling only from the central regions of feature maps eliminated both the intensity arm and the shared activity pattern seen in the decoding trajectories (see Supplemental Fig. 16). Although these artifacts distort the representation—indeed, the smoothness of the intensity arm reflects how padding-related information propagates across feature maps—they are part of the network's normal operation. Excluding them would therefore misrepresent the model's true internal dynamics, so we retained them in our manifold analysis.

A.14. Software

All software (Table 2) is used in accordance with its respective license.

A.15. Compute

The experiments were conducted on an HPC cluster. FNN sampling uses randomly selected GPUs (RTX 2080 Ti, or better). All other experiments were performed on CPU. All experiments required less than 30 GB memory. In total, 10 tensor decomposition experiments were run on CPU, each taking 2 days on a single CPU. Preliminary results not included in the paper required another 50 tensor decomposition experiments.

A.16. Language model usage

At the level of individual words or partial sentences, language models were used to fix language errors. Minor code sections were produced by language models and used only after careful inspection.

Table 2. Software packages used in this work.

**Package**	**Version**	**License**
MATLAB Tensor Toolbox (Bader et al., 2023)	3.6	BSD-2
IAN (Dyballa & Zucker, 2023)	1.1.2	BSD-3
NeuralEncodingManifolds (Dyballa et al., 2024a)	N/A	BSD-2
NumPy (Harris et al., 2020)	1.25.0	BSD-3
SciPy (Virtanen et al., 2020)	1.15.3	BSD-3
scikit-learn (Pedregosa et al., 2011)	1.7.1	BSD-3
PyTorch (Paszke et al., 2019)	2.6.0	MIT
Matplotlib (Hunter, 2007)	3.10.1	PSF-based (BSD-compatible)
Plotly (Inc., 2015)	6.0.0	MIT
TUEplots (Krämer et al., 2024)	0.2.0	MIT

B. Data and code availability

Upon acceptance, we will publish a GitHub repository with the full code necessary to reproduce all experiments and figures in this paper. We will also provide rotating video animations of three-dimensional visualizations to aid interpretation.

C. Supplemental Tables and Figures

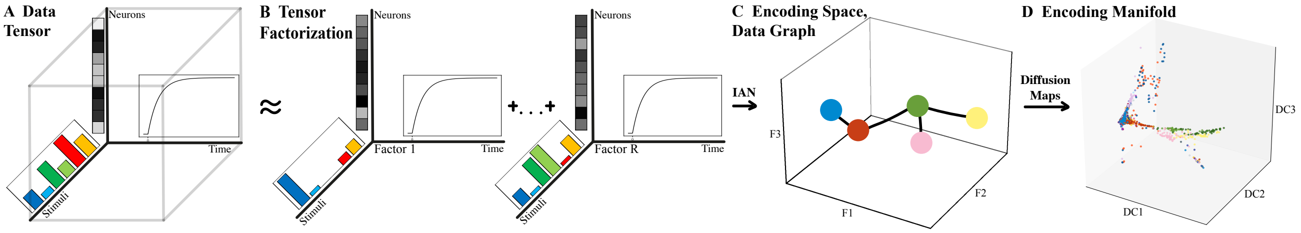

Figure 6. **Encoding Manifold Pipeline (A, B)** A non-negative tensor factorization of the original data tensor identifies those neural factors that account for part of the stimulus ensemble over comparable time epochs. **(C)** Collecting the neural factors into a linear vector space, an adaptive-neighborhood kernel builds a data graph. **(D)** Diffusion maps yield the encoding manifold.

Table 3. Stimulus classification accuracy for Leave-One-Out 3-Nearest Neighbor (3-NN) and Logistic Regression (LR) classifiers trained on each layer’s activations. Methods in Appendix A.

Accuracy	L1	L2	L4	L5	L7	L8	Rec	RecOut	Readout	Out
LR	0.59	0.62	0.66	0.65	0.71	0.74	0.89	**0.90**	0.88	0.77
3-NN	0.41	0.66	0.58	0.52	0.53	0.61	**0.73**	0.64	0.63	0.67

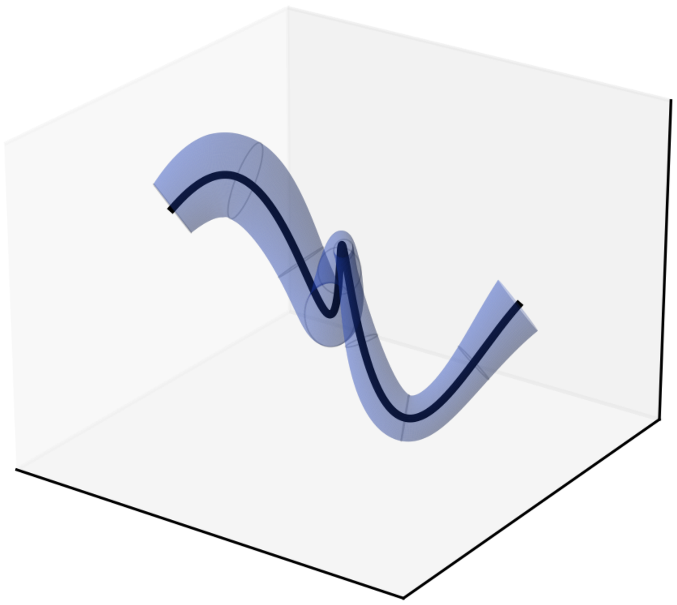

Figure 7. A tubular neighborhood around a centerline $c(u)$ with radius profile $R(u)$.

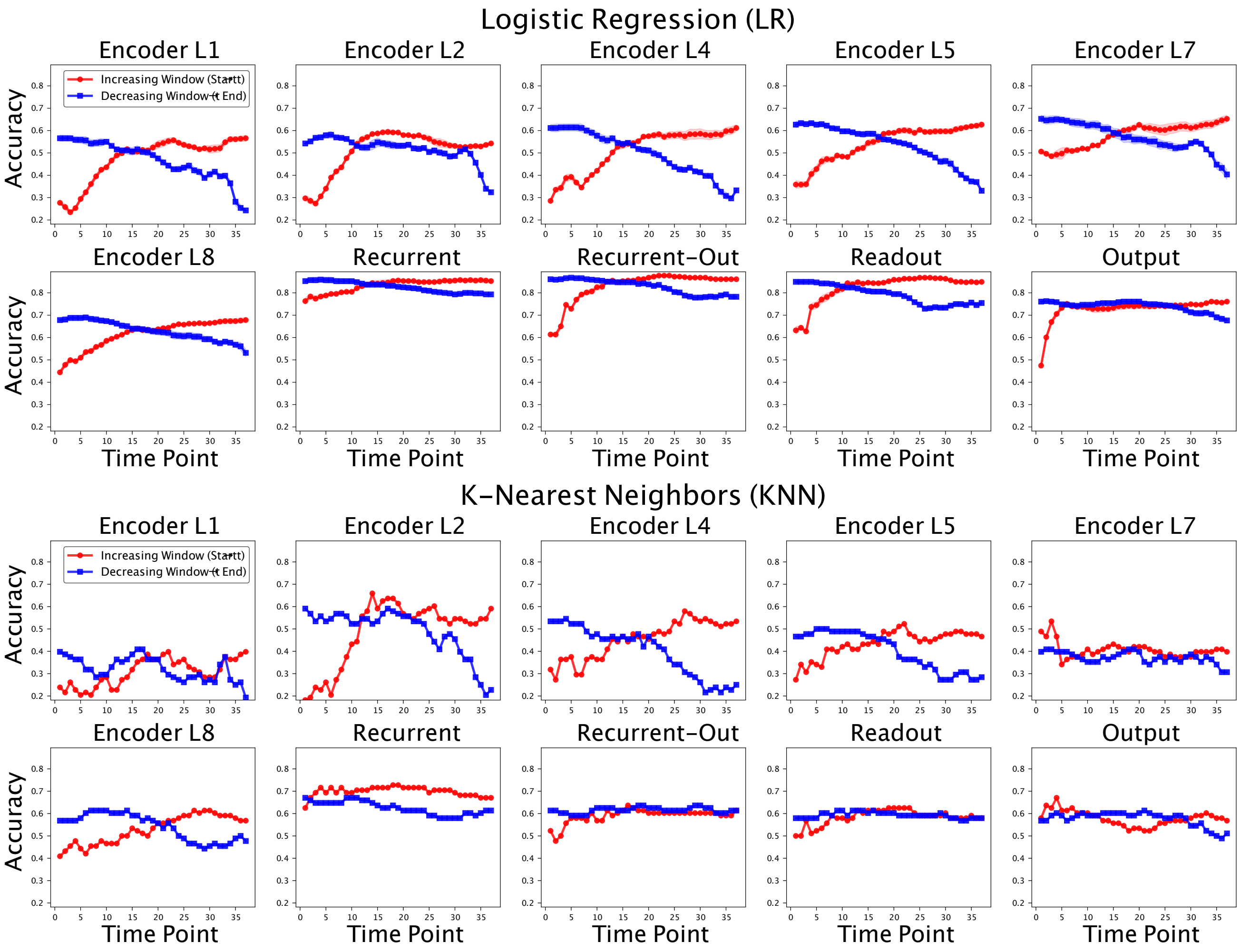

Figure 8. **Logistic regression (LR, top) and K-Nearest Neighbor (KNN, K=3, bottom) classifier accuracy** for each layer. We use increasing time windows (timesteps 0 → t, red) or decreasing time windows (t → 37, blue) to calculate the accuracies. Shaded regions for LR show the s.e.m. The maxima across panels are summarized in Table 3.

Table 4. **Tubularity metrics for biological and FNN data**. Low tightness and crossings values indicate high tubularity. The biological trajectories show highly tubular organizations compared to FNN. Method details in Appendix.

Layer	Ground Truth Labels		HDBSCAN Labels		
	S_{tight}	S_{cross}	S_{tight}	S_{cross}	Clusters
Retina	0.0688	1.29×10^{-05}	0.1017	1.06×10^{-05}	4
V1	0.1357	4.06×10^{-05}	0.1859	3.50×10^{-05}	4
Enc1	0.2018	2.87×10^{-04}	0.7680	1.66×10^{-04}	1
Enc13	1.9885	1.77×10^{-06}	4.3461	1.09×10^{-06}	3
Rec	0.1228	2.65×10^{-07}	0.1697	1.53×10^{-07}	4
RecOut	0.1209	5.72×10^{-07}	0.1650	5.34×10^{-07}	4
Readout	0.3307	3.96×10^{-06}	0.4320	5.40×10^{-06}	4
Output	0.1483	3.53×10^{-06}	0.2784	1.12×10^{-06}	3

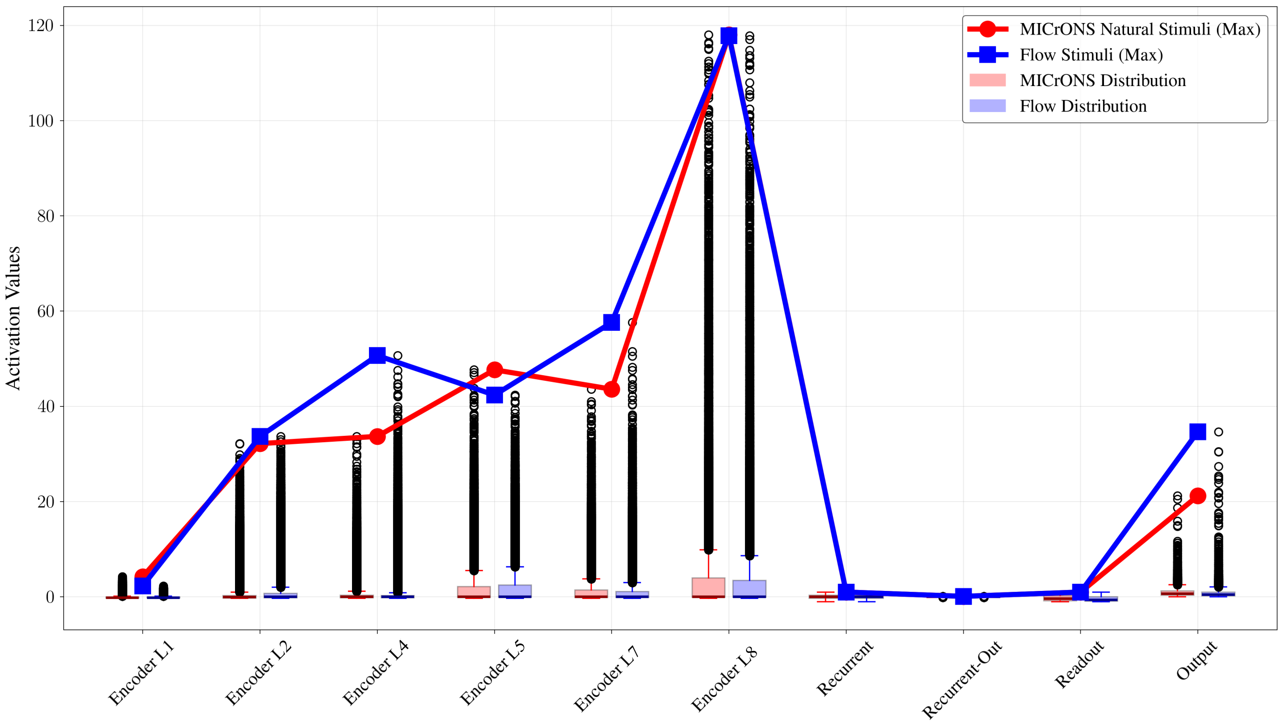

Figure 9. **Activation function output distributions and maxima** for natural MICrONS (Bae et al., 2025) input videos and the flow stimulus ensemble (Dyballa et al., 2024a). The comparable activity across network layers shows the adequacy of investigating the FNN with flow stimuli. The differences in magnitudes across layers are explained by the activations functions (GELU in the *encoder*, Tanh in the *recurrent* and *readout* modules).

Table 5. Representational Similarity Analysis (RSA), Canonical Correlation Analysis (CCA), Linear Predictivity (LP) and Dynamic Similarity Analysis (DSA) scores and DSA Z-scores to a time-shuffled baseline. High values indicate closer alignment for all metrics. (see Appendix A.12)

Region	Metric	L1	L2	L4	L5	L7	L8	Rec	Readout	Output
Retina	RSA	-.04	-.03	-.03	-.01	-.01	0.03	0.03	**0.05**	-.01
Retina	CCA	0.19	0.25	0.25	0.30	0.27	0.26	**0.34**	0.25	0.32
Retina	LP	0.05	0.06	0.17	0.29	0.26	0.28	**0.43**	0.24	0.26
Retina	DSA	**0.84**	0.77	0.83	0.73	0.58	0.56	0.80	0.81	0.80
Retina	DSA-Z	**5.90**	4.77	5.29	4.55	2.35	1.87	2.00	1.79	4.45
V1	RSA	-.11	-.27	-.16	-.22	0.10	0.08	**0.46**	0.08	0.41
V1	CCA	0.29	0.31	0.33	**0.39**	0.29	0.35	**0.39**	0.29	0.38
V1	LP	0.05	0.04	0.18	0.29	0.23	0.27	**0.40**	0.22	0.24
V1	DSA	0.91	0.76	**0.93**	0.76	0.59	0.57	0.88	0.92	0.90
V1	DSA-Z	**7.03**	5.49	6.52	5.91	5.46	4.40	4.72	3.50	5.27

Table 6. Explained Variance (EV, in %) of decoding manifold PCA, and number of tensors (R) and Error Percentage (EP) of tensor factorization (in %)

Metric	L1	L2	L4	L5	L7	L8	Rec	RecOut	Readout	Output
EV	47.91	57.66	59.52	48.20	48.53	42.77	57.73	53.43	53.99	55.23
R	8	12	11	11	13	11	9	12	17	14
EP	37.08	25.50	23.02	23.35	22.34	23.51	15.68	16.45	13.81	9.26

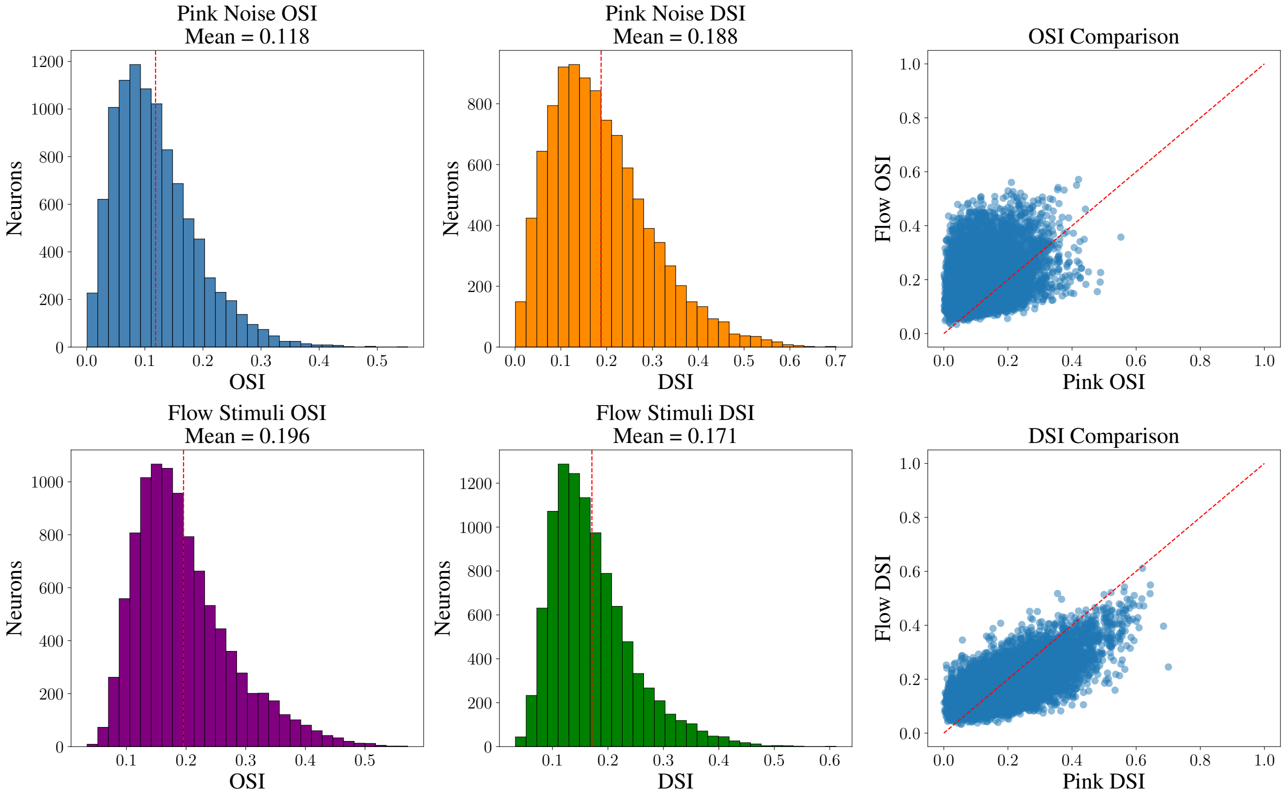

Figure 10. **OSI and DSI of FNN output** for pink noise (as used in Wang et al. (2025)) and for the stimulus ensemble from Dyballa et al. (2024a), meaned over the different stimuli.

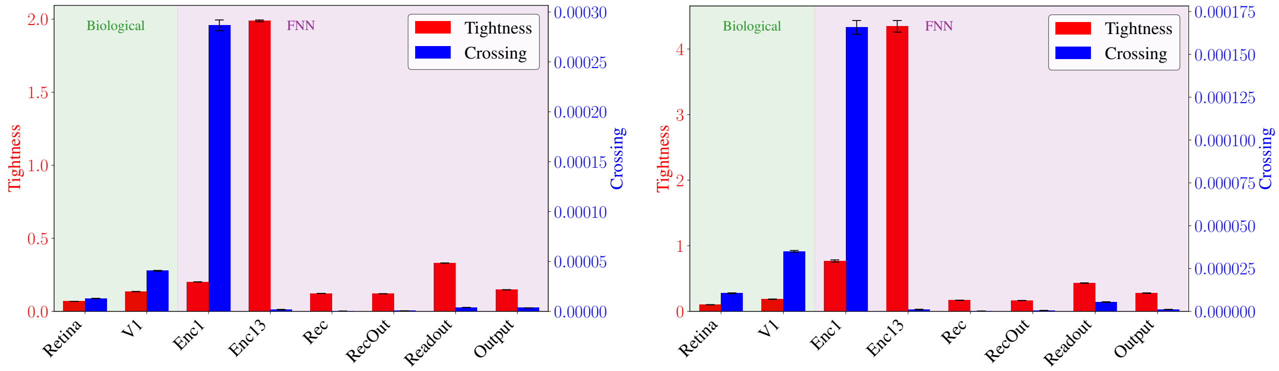

Figure 11. **Tubularity comparison between biological and FNN data.** Tightness, measuring how close trajectories within a bundle are to their centerline, and crossings, measuring the amount of transverse crossings in a bundle, are scores for biological and FNN data. Left: Using ground-truth stimulus class labels. Right: Using HDBSCAN (Campello et al., 2013) labels.

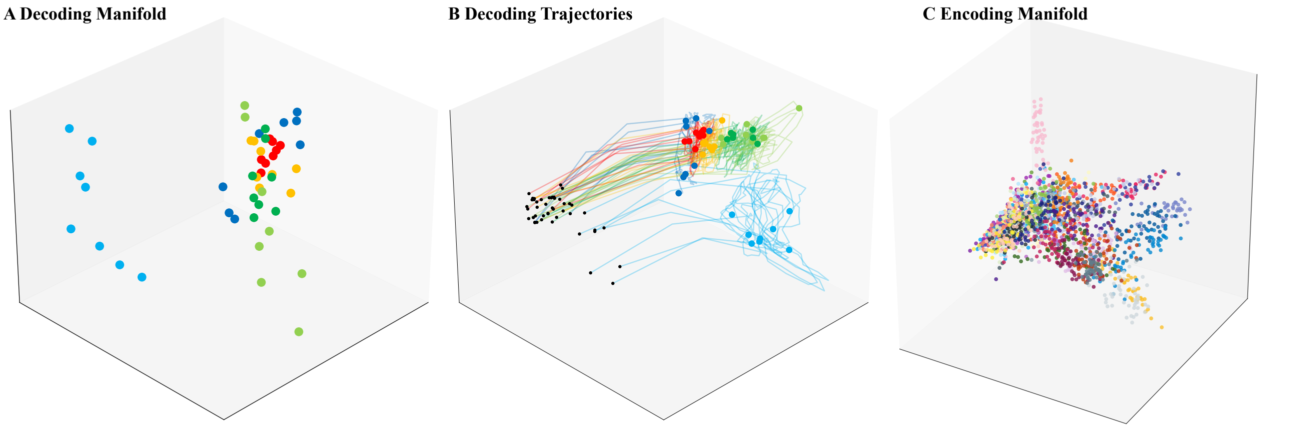

Figure 12. **Encoder L2.** Note the encoding manifold smoothness here results from the early layer only capturing simple features and the dominance of intensity discussed for L8. We therefore do not interpret this as a V1-like smooth encoding manifold.

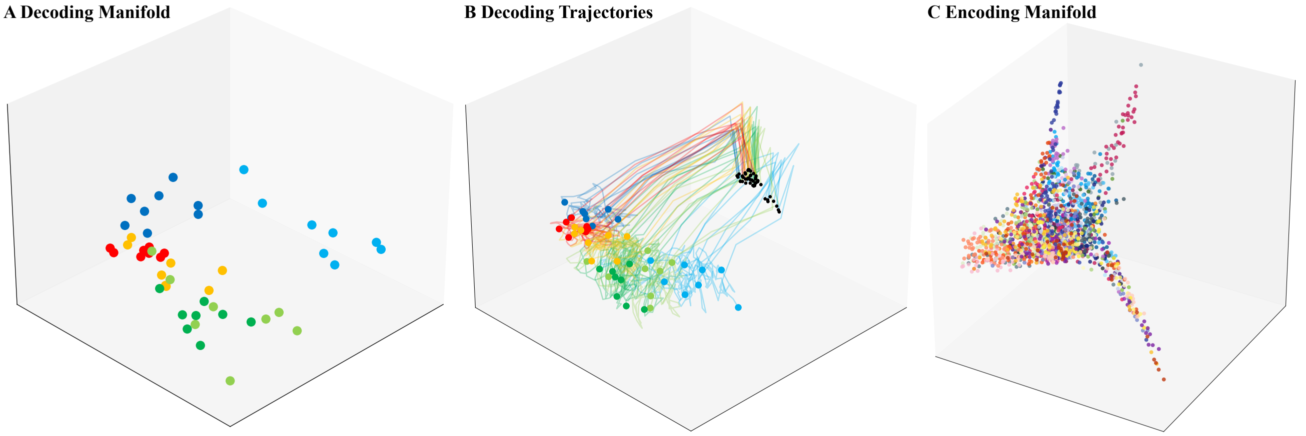

Figure 13. **Encoder L4.** Note the encoding manifold smoothness here results from the early layer only capturing simple features and the dominance of intensity discussed for L8. We therefore do not interpret this as a V1-like smooth encoding manifold.

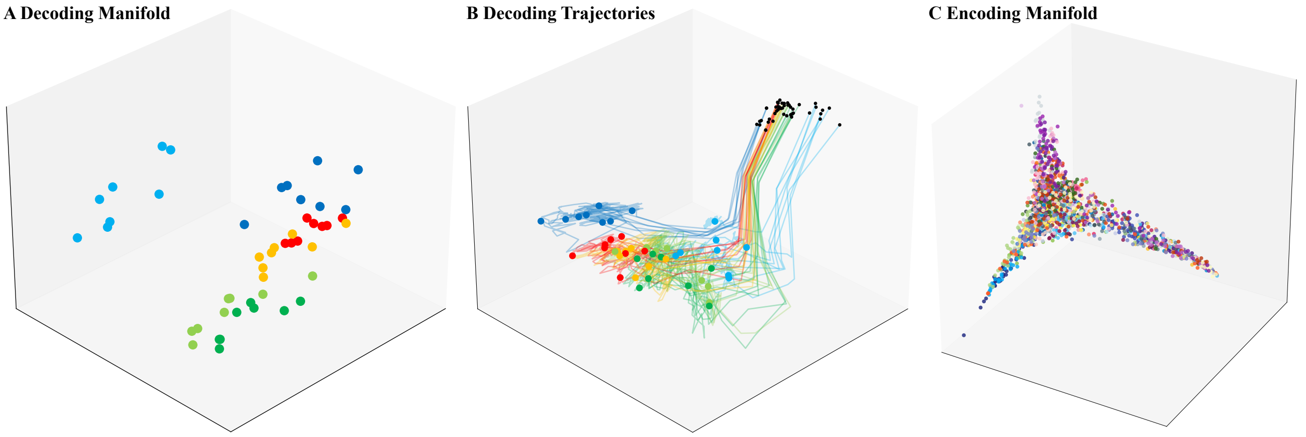

Figure 14. **Encoder L5.** Note the encoding manifold smoothness here results from the early layer only capturing simple features and the dominance of intensity discussed for L8. We therefore do not interpret this as a V1-like smooth encoding manifold.

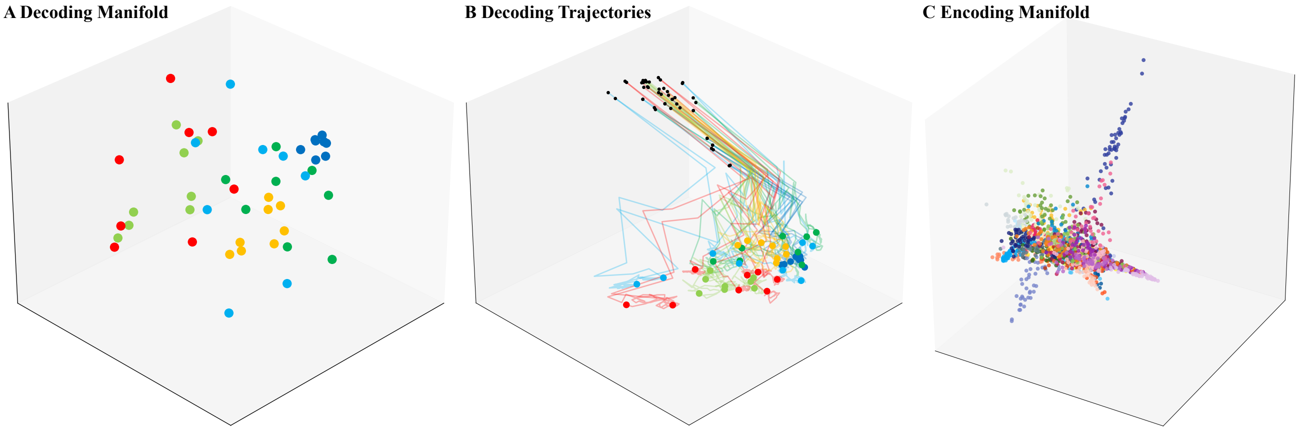

Figure 15. **Encoder L7**

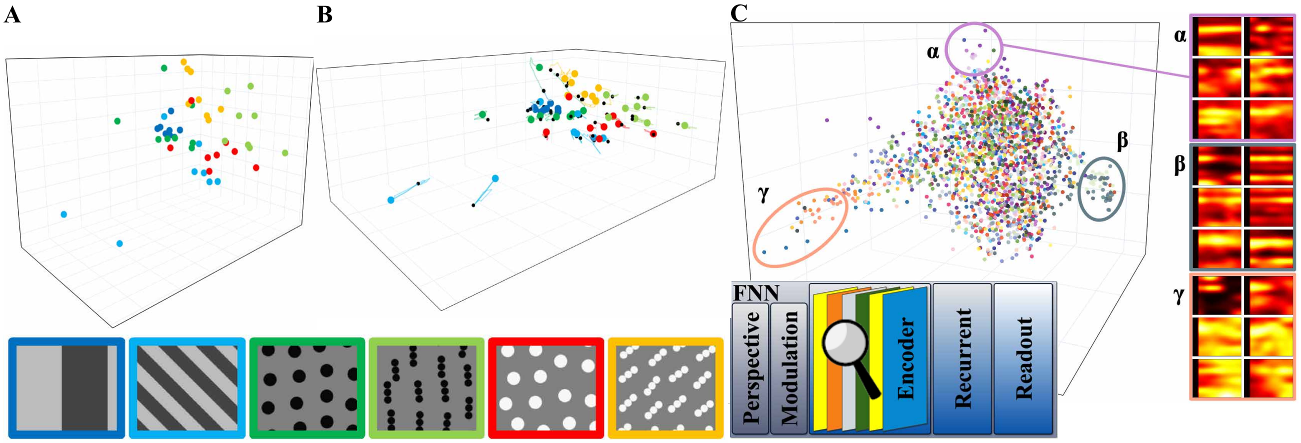

Figure 16. **Encoder L8 decoding manifold, trajectories and encoding manifold without intensity artifacts**. Without the intensity artifacts there is no temporal development at all in the decoding trajectories (comparable to encoder L1) apart from the jump after the 0-th step. The non-selective high intensity neurons are padding artifacts at the edges of the image. In the encoder, due to spatial convolutions, the effect of these artifacts spreads out across the feature maps. This is supported by the intensity smoothly organizing the manifold with a transition from intensity-only neurons to selective responses. In the recurrent stage, the function of the attention layer is capable of filtering exactly those artifacts out. The artifacts are reintroduced by the recurrent-output convolution, but then filtered out by the readout interpolation from central neurons only.

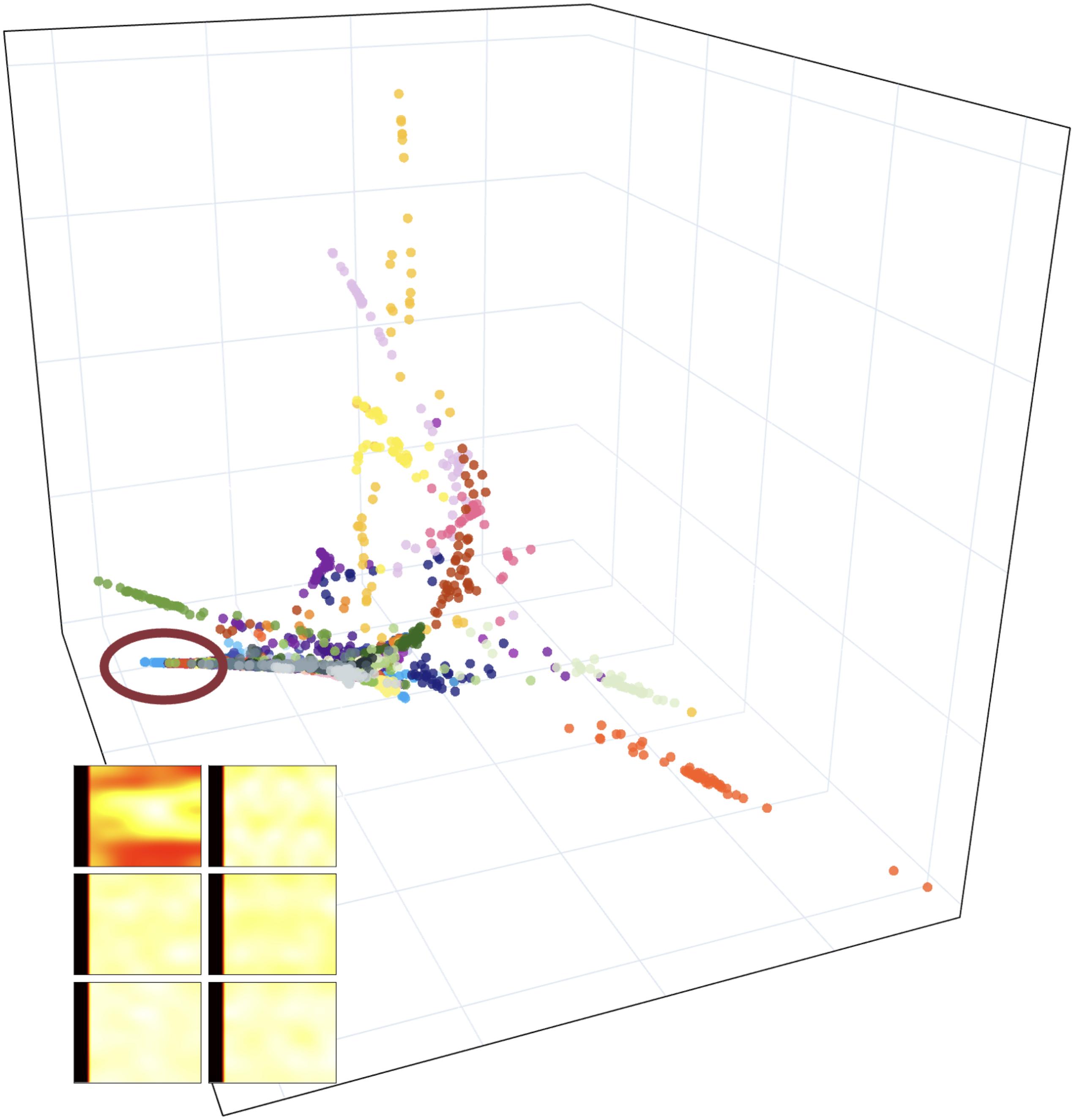

Figure 17. **Minimodel encoding manifold with intensity arm**. The intensity artifacts are also present in the border regions of feature maps in the model from Du et al. (2025).

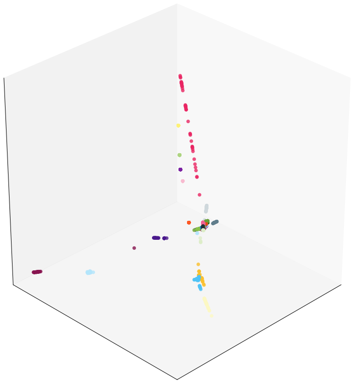

a) 2000 Neurons, 40 feature maps, 50 neurons each, intensity-based feature map and neuron sampling.

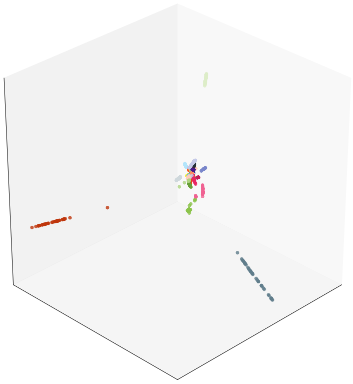

b) 2000 Neurons, 40 feature maps, 50 neurons each, random feature map, and intensity-based neuron sampling.

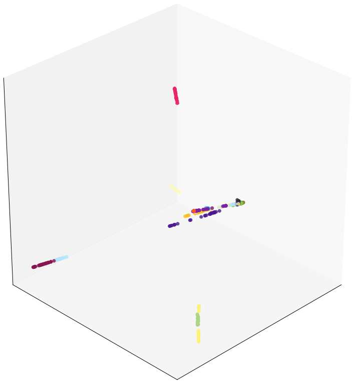

c) 2000 Neurons, 40 feature maps, 50 neurons each, intensity-based feature map, and random neuron sampling.

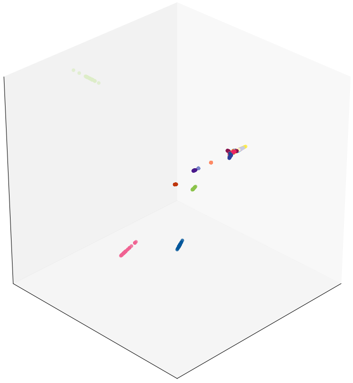

d) 2000 Neurons, 40 feature maps, 50 neurons each, fully random feature map and neuron sampling.

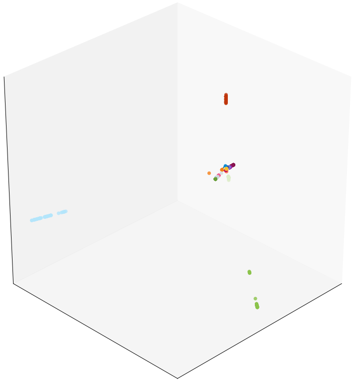

e) 4000 Neurons, 80 feature maps, 50 neurons each, intensity-based feature map and neuron sampling.

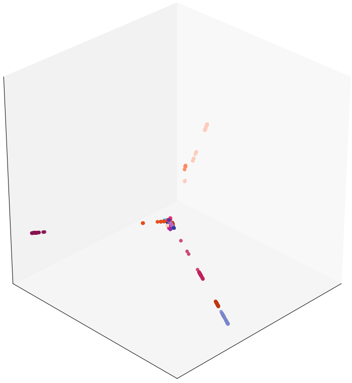

f) 5000 Neurons, 50 feature maps, 100 neurons each, intensity-based feature map and neuron sampling.

Figure 18. **Sampling tests for readout encoding manifolds**. The encoding manifold for all sampling conditions look similar, having clusters by feature maps.
